# The Story of the Insane from Year to Year

**Published:** 1893-05-13

**Authors:** 


### THE STORY OF THE INSANE FROM YEAR TO YEAR.

(Fifth Series.)

### WARWICK COUNTY ASYLUM.

On January 1st the numbers in this asylum were 645, and by the end of December they were 698. There were 208 admissions, 68 recoveries, and 59 deaths. The percentage of recoveries is 32·6, and that of the deaths is 8·8. Intemperance caused the insanity in 28 instances, and heredity in 47. Six of the deaths were caused by consumption and dysentery; enteric fever and diarrhœa collectively account for 6. Post-mortem examinations were made in 53 out of the 59 deaths, a proportion which we should feel inclined to say was considerably too high. Although this wholesale making of post-mortem examinations may enable Dr. Miller to confirm his diagnosis, it does not seem to help much in the treatment of the living, for we find him saying he "cannot report any great advancement in the medical treatment of the patients." Further accommodation would seem to be urgently required. The female division is overfull, and there are only 19 vacancies on the male side, while 57 patients are boarded out at the Birmingham Asylum. Dr. Miller thinks the asylum might be increased to 850 beds, and he is, we are pleased to note, opposed to large asylums, thinking that "the capabilities and energy of a man of ordinary calibre will be sorely strained when he is asked to administer to the requirements of a population exceeding 1,000." There is a balance against the farm of £31; but rent of land and patients' labour are included in the accounts. The cost of maintenance is 9s. 3d. per head per week.

### OMAGH DISTRICT ASYLUM.

This institution receives the patients chargeable to the counties of Fermanagh and Tyrone. Table I. tells us that the limit of accommodation is 510 beds; but it also tells us that there were in residence at the beginning of the year 561 patients, and that by the end of the year the number had risen to 565, so there must have been considerable overcrowding throughout the year. The admissions were 154; the recoveries 79, and the deaths 36. The recoveries, calculated on the admissions, reach the creditable percentage of 51·3, and the death rate is very low, being only 6·4 per cent. on the average number resident. Intemperance does not seem to be a common cause of insanity in Fermanagh and Tyrone, as only five of the total admissions are put down to it; but as not fewer than 81 are said to be of unknown causation it is clear that no reliable deduction can be drawn from the table. Hereditary influence accounts for 27 cases. Eight of the deaths, being close on 25 per cent. of the whole, were due to pulmonary consumption. It would be interesting to know whether this is attributable to the over-crowding. Dr. Carre's report has the merit of shortness. We are pleased to note that "no form of mechanical restraint was used during the year, and no patient was placed in seclusion." Not that we have any objections to these forms of treatment—far from it—but it is satisfactory that out of 715 patients no one required either the one or the other. The cost per head per annum is £22 6s.

### NORFOLK COUNTY ASYLUM.

On January 1st, 1891, this asylum contained 687 patients, and on the last day of the year 740 were in residence. One hundred and ninety-seven patients were admitted; 66 were discharged recovered; and 64 died. Thrown into percentages in the manner usual in asylums we find that the recoveries come out at 41·77 per cent., and the deaths at 8·8. The recoveries are about as much above the average in English county asylums as the deaths are below it. As to the causes of the insanity in the 197 cases admitted we notice that hereditary influence accounts for 30 per cent., and intemperance in drink for 13 cases. It is creditable to Dr. Thomson that in only 27 instances is the cause unknown, for we know how difficult it is to extract information from friends, and how careless union authorities often are in filling up the answers to these questions. Pulmonary consumption caused death in 11 cases, being about 17 per cent., of the whole. It is always a pleasure to run through Dr. Thomson's report; it is so well arranged in form, and so clear in diction. We notice he has had a visit from that troublesome disease, dysenteric diarrhœa, which has lately been going the round of our asylums, new and old, and the cause of which has so far baffled most of the medical superintendents. It would almost seem as if a new disease had been developed during the last decade or two. Dr. Thomson tried vegetarian diet in epilepsy, and he tells us that "speaking generally, neither the frequency nor severity of the fits was diminished by the abstinence from butcher's meat." This is the opposite conclusion to that which Dr. Merson came to. Out of 24 cases experimented on by this able physician there was in 14 a decided decrease in the number of fits. Dr. Thomson says that "with the increasing and vexatious clerking duties thrown upon medical superintendents by the recent Lunacy

Acts and Lunacy Commissioners' regulations, the treatment of patients, which I understand to be their primary functions, cannot help being in a greater measure delegated to their less experienced colleagues, the assistant medical officers; a more ill-advised and uncalled-for piece of legislation, not to speak of the almost unintelligible English in which the section is worded, than the annual, biennial, triennial and quinquennial recertification of chronic cases it would be difficult to find." Attendant William Thompson was voted a gratuity of £5, for jumping into the river and saving a patient's life at the risk of his own. The attendant was also awarded the Royal Humane Society certificate. It is a pity that acts of bravery and sacrifice are not more often recognized and rewarded. Few people need more sympathy and get less, by the public at least, than asylum attendants and nurses. The Norfolk Asylum is evidently marching on under Dr. Thomson's superintendence. Very many improvements have been carried out during the year. The visitors say they have pleasure in recording their satisfaction with the efficient manner in which he has managed the establishment.

